# The Effects of Lycopene and Insulin on Histological Changes and the Expression Level of Bcl-2 Family Genes in the Hippocampus of Streptozotocin-Induced Diabetic Rats

**DOI:** 10.1155/2017/4650939

**Published:** 2017-06-01

**Authors:** Masoume Soleymaninejad, Seyed Gholamali Joursaraei, Farideh Feizi, Iraj Jafari Anarkooli

**Affiliations:** ^1^Department of Anatomy, School of Medicine, Babol University of Medical Sciences, Babol, Iran; ^2^Department of Anatomy, School of Medicine, Zanjan University of Medical Sciences (ZUMS), Zanjan, Iran

## Abstract

The aim of this study was to evaluate the effects of antioxidants lycopene and insulin on histological changes and expression of *Bcl-2* family genes in the hippocampus of streptozotocin-induced type 1 diabetic rats. Forty-eight Wistar rats were divided into six groups of control (C), control treated with lycopene (CL), diabetic (D), diabetic treated with insulin (DI), diabetic treated with lycopene (DL), and diabetic treated with insulin and lycopene (DIL). Diabetes was induced by an injection of streptozotocin (60 mg/kg, IP), lycopene (4 mg/kg/day) was given to the lycopene treated groups as gavages, and insulin (Sc, 1-2 U/kg/day) was injected to the groups treated with insulin. The number of hippocampus neurons undergoing cell death in group D had significant differences with groups C and DIL (*p* < 0.001). Furthermore, insulin and lycopene alone or together reduced the expression of *Bax*, but increased *Bcl-2* and *Bcl-x_L_* levels in DI, DL, and DIL rats, especially when compared to group D (*p* < 0.001). The ratios of *Bax/Bcl-2* and *Bax/Bcl-xL* in DI, DL, and DIL rats were also reduced (*p* < 0.001). Our results indicate that treatment with insulin and/or lycopene contribute to the prevention of cell death by reducing the expression of proapoptotic genes and increasing the expression of antiapoptotic genes in the hippocampus.

## 1. Introduction

Several studies have been conducted in conjunction with nervous disorders caused by diabetes and have primarily focused on disorders of the peripheral nervous system. However, recent research has also been implemented on the central nervous system in people with diabetes [1–3]. In the recent years, it has been found that the central nervous system attains various functional problems such as electrophysiological issues, cognitive disorders, and structural changes to the hippocampus, which are imperative for learning and memory [4, 5]. Hence, the hippocampus is sensitive to increased levels of blood glucose and has a greater level of damaged neurons in type 1 diabetes [6, 7].

In STZ-induced diabetic rats, the hippocampus is significantly affected, thereby causing cognitive deficits [8]. As a result, cognitive disorders, particularly impaired memory and learning in type 1 diabetes, are continuing to increase dramatically. As diabetes greatly increases the death of neurons in the hippocampus, the loss of neurons due to apoptosis has become one of the main known reasons for dysfunction in diabetic rats [9].

Recent research findings demonstrated that nerve cell death is responsible for histopathological changes and pathophysiological disorders related to the central nervous system [10]. Additionally, several studies have introduced neuronal death in the hippocampus of rats with STZ-induced diabetes. Apoptosis, more specifically, is a form of cell death characterized by the fragmentation of DNA in the cell nucleus [5, 11, 12]. Although many factors are involved in the apoptotic process, the key elements are limited to two groups, including caspases and the *Bcl-2* family genes [13]. The *Bcl-2* family, for example, are involved in apoptosis and is the second category divided into two main groups of inhibitors (the head of Bcl-2) and inducers of apoptosis (with the head of *Bax*) [14].

It seems that the pathophysiological changes in diabetes can be caused by increased levels of free radicals [15]. Free radicals play an important role in many physiological and biochemical processes of the human body [16]. High levels of free radicals, however, and simultaneous reductions in antioxidant defense mechanisms can, therefore, lead to cellular organelles and enzymes becoming damaged as a result of oxidative stress [10]. Reactive oxygen species (ROS), on the other hand, increases both type 1 and type 2 diabetes. Thus, sources of oxidative stress in diabetes may include the auto-oxidation of glucose, a reduction in the tissue concentration and molecular weight of antioxidants, and impairments in the antioxidant defense enzyme activity [16]. The central nervous system and brain are very vulnerable to free radicals, and it is the antioxidants which donate unpaired electrons of free radicals to neutralize their effects [17]. In healthy people, there is a balance between free radicals and antioxidants. However, it has been shown that people with diabetes have higher levels of free radicals which can cause complications in diabetes. Studies have shown that treatment using antioxidants can reduce some of the complications of diabetes [18]. Studies have shown that treatment with antioxidants can lessen complications of diabetes. Therefore, antioxidants are important in reducing the level of ROS. Lycopene, for instance, is one of the most powerful antioxidants primarily found in red fruits and vegetables, such as watermelon, tomatoes, and grapes; it also has higher antioxidant activity than beta-carotene [19]. Lycopene is a carotenoid with Nan-pro-vitamin A and 11 double linear bonds [10, 19].

Medicines are primarily used to prevent, diagnose, or treat variety of diseases or chronic disorders. However, they should be used with caution to ensure they are safe and effective [20]. In a broad survey regarding food sources containing lycopene, it was found that lycopene has both antioxidant and antidiabetic activity [21, 22]. Despite this, the antiapoptotic role of lycopene has little information available. Hence, this study is going to overview the protective effect of insulin and lycopene (as an antioxidant), both alone and simultaneously, on histopathology and expression level of Bcl-2 family gene in the hippocampus of STZ-induced diabetic rats.

## 2. Method and Materials

The present experimental study was carried out on male Wistar rats. 48 male Wistar rats weighing approximately 200–250 g, aged between 8 and 10 weeks old, were attained from the animal house of Baqiyatallah-Allah from Tehran University of Medical Sciences. All rats were purchased and moved to the animals' house at Zanjan University of Medical Sciences. After a week of maintenance, to comply with the conditions of the Animal House environment, the rat's weight was recorded and their blood glucose was measured using a glucometer (Accu-Chek, Germany). Diabetic rats after 24 hours of fasting were induced using STZ (Sigma, USA) and a single intraperitoneal (IP) injection, at 60 mg/kg. For the injection, STZ was solved in a citrate buffer at 1.0 M (pH = 4.5) and was used at 8 am on the target groups while in their fasting state.

Three days after the administration of STZ, blood glucose in diabetic rats was measured and recorded. The criterion for being diabetic was considered higher than 250 mg/dl regarding blood glucose levels [23]. Any rats with blood glucose lower than 250 mg/dl were excluded. The diabetic rats were then randomly divided into four groups of eight as follows:

1. The diabetic group (D) that did not receive anything (untreated diabetic group)
2. The diabetic group treated with insulin (DI) and received protamine (NPH) (pharmacy elixir of Borujerd, Iran) at a rate of two units a day by subcutaneous injection (SC)
3. The diabetic group treated with lycopene (DL) at a rate of 4 mg per kg of body weight (dissolved in distilled water distilled twice), which was administrated as gavages [24]
4. The diabetic rats treated with insulin and lycopene (DIL) who received insulin and lycopene as well as the DL and DI groups. In addition to the diabetic groups, nondiabetic rats were randomly divided into two groups known as the control (C) and the lycopene control (CL) groups.

Groups C and D received normal saline only. The above treatments were continued for eight weeks, and at the end of the treatment period, rats were weighed, and blood glucose levels were measured. Following this, the animals were anesthetized using chloroform and have their brains removed. The hippocampus was also further removed from the brain after detaching the meninges curtain. In order to evaluate the histological changes, one side of the hippocampus was fixed in 10% formalin and the other side of the hippocampus was stored at −70°C for RNA extraction.

### 2.1. Histopathological Study

Hippocampus obtained from rats studied for 72 hours was placed in 10% formalin and was followed by tissue processing performed on samples of the hippocampus. Following this, a rotary microtome from 5 *μ*m thick sections were prepared and stained with hematoxylin-eosin (H&E). Finally, sections were pictured using a light microscope (Olympus BX51) equipped with a camera, at a magnification of 40x. Overall, 10 sections were randomly chosen from each rat, and hippocampal neurons were selected to count for the number of healthy and unhealthy cells in the areas of CA1, CA2, CA3, and DG. Healthy neurons with clear cytoplasm and a round nucleus were distinguished from the others. Dark neurons with a dense, irregular, and wrinkled nucleus were considered as dead neurons.

### 2.2. Reverse Transcriptase Polymerase Chain Reaction (RT-PCR)

Hippocampal total RNA was isolated from tissue samples using a TriPure Isolation Reagent according to the protocol, and absorbance of RNA extracted was measured using the optical density of the NanoDrop 2000 device (Thermo Scientific). In order to determine the amount of mRNAinBcl-2, Bcl-x_L_, and Bax genes, semiquantitative RT-PCR reactions were performed. For implementation of PCR (AccuPower RocketScript RT-PCR Premix), a Bioneer company kit was used. The finalized RT-PCR mixture (final volume of 20 ml) contained 2 ml mRNA, 10 pmol of each gene-specific primers (Bcl-2, Bcl-x_L_, and Bax genes), and 10 pmol of GAPDH gene specific primers (used as the internal control) (Table 1). Data related to the expression of mRNA for Bcl-2, Bcl-x_L_, and Bax was measured using the semiquantitative RT-PCR method. For this purpose, the ImageJ software was attained and bands of the electrophoresis of PCR products were evaluated using densitometry. Quantitative data for each of the three genes were normalized with quantitative data related to GAPDH as an internal control.

### 2.3. Statistical Analyses

All data were analyzed by the Kruskal-Wallis nonparametric analysis of variance (ANOVA), followed by the Mann–Whitney *U* test. The latter test was also used where two groups were compared with each other. For apoptosis analysis, the counts were expressed as mean ± SEM. In all statistical evaluations, *p* < 0.05 was considered as the criterion for statistical significance.

## 3. Results

### 3.1. Blood Glucose and Body Weight Changes

At the beginning of the study, no significant difference was observed for the weight of rats in all groups. After eight weeks, however, the weight of diabetic rats (D) was found to be reduced significantly, when compared with that of the control group (C) (*p* < 0.001). In addition, at the end of the treatment period, the weight of diabetic rats treated with insulin (DI), and insulin and lycopene (DIL), was increased significantly when compared with that of group D (*p* < 0.01) (Table 2).

Furthermore, blood glucose levels in diabetic rats treated with insulin (DI), and insulin and lycopene (DIL) when compared to those in nontreated diabetic rats (D), was substantially reduced (*p* = 0.01). As well as this, blood sugar levels of diabetic rats treated with lycopene (DL) were significantly reduced when compared to those of the on-treated diabetic group (D) (*p* < 0.05). No significant differences in the blood glucose levels of DI, DL, DIL, and C were observed (Table 2).

### 3.2. Histopathological Assessment

**Figure 1:**
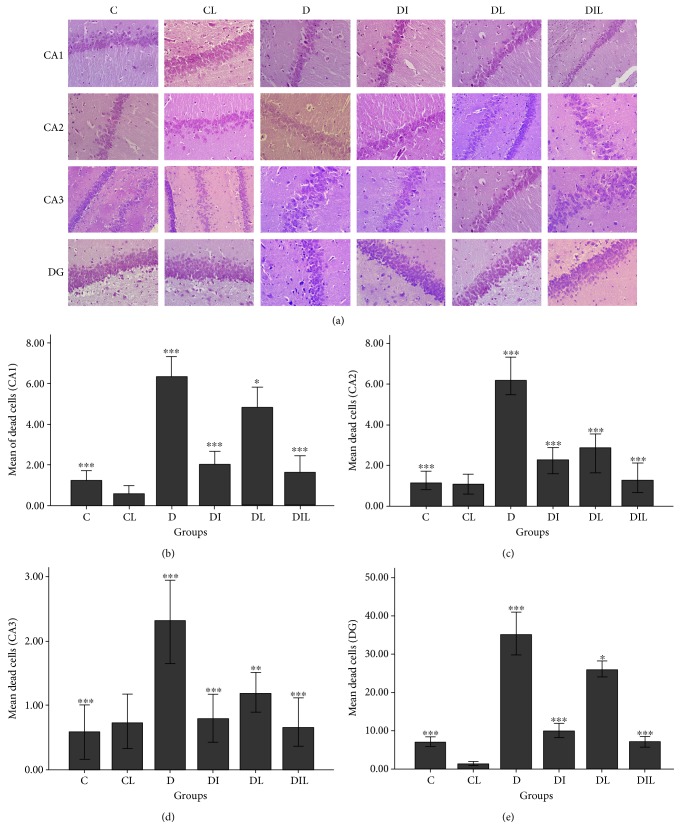
The effects of insulin and lycopene on neural cell death of hippocampus. (a) Neuronal cell death was evaluated via hematoxylin-eosin staining at 40x magnification. C: the control group; CL: the control group treated with lycopene; D: untreated diabetic group; DI: diabetic group treated with insulin; DL: diabetic group treated with honey; DIL: diabetic group treated with insulin and honey simultaneously. (b) ^∗∗∗^*p* < 0.001, comparing C, DI, and DIL with D. ^∗^*p* < 0.05, comparing D with DL. (c) ^∗∗∗^*p* < 0.001, comparing C, DI, DL, and DIL with D. (d) ^∗∗∗^*p* < 0.001, comparing C, DI, and DIL with D. ^∗^*p* < 0.01, comparing D with DL. (e) ^∗∗∗^*p* < 0.001, comparing C, DI, and DIL with D. ^∗^*p* < 0.05, comparing D with DL. Bar graphs indicate the mean ± SEM (*N* = 8).

The hematoxylin-eosin staining method was used for staining the nucleus and cytoplasm. In H&E staining, nuclei are stained blue and the cytoplasm is marked as pink. Normal cells with eosinophilic light cytoplasm and nuclei are clear. Dead cells are recognized with a dark cytoplasm or undetectable nuclei, whereas dead cells seem to be smaller in size than normal eosinophilic cytoplasm cells and are determined by the pyknotic nucleus (Figure 1(a)).

Counting the number of normal cells in CA1, CA2, and DG areas of the studied rats, there was no established significant difference. In terms of the number of normal cells in the CA3 region, none of the groups studied showed a significant difference. Instead, the studied groups showed a significant difference in terms of the number of dead cells for different areas of the hippocampus. Dead cell population in the hippocampus (different areas) of rats for the diabetic group with no treatment (D) was also significantly lower than that for the control group (C), the insulin-treated diabetic group (DI), and the diabetic rats treated with insulin and lycopene simultaneously (DIL) (*p* < 0.001). Diabetic rats treated with lycopene (DL) similarly had a lower number of dead cells than in the different regions of hippocampus when compared to the diabetic group without treatment (D) (*p* < 0.05 in the CA1 and DG, *p* < 0.01 in the CA3, and *p* < 0.001 in the CA2) (Figures 1(b), 1(c), 1(d), and 1(e)).

### 3.3. Expression of Bax, Bcl-2, and Bcl-x_L_ Genes at mRNA Level

**Figure 2:**
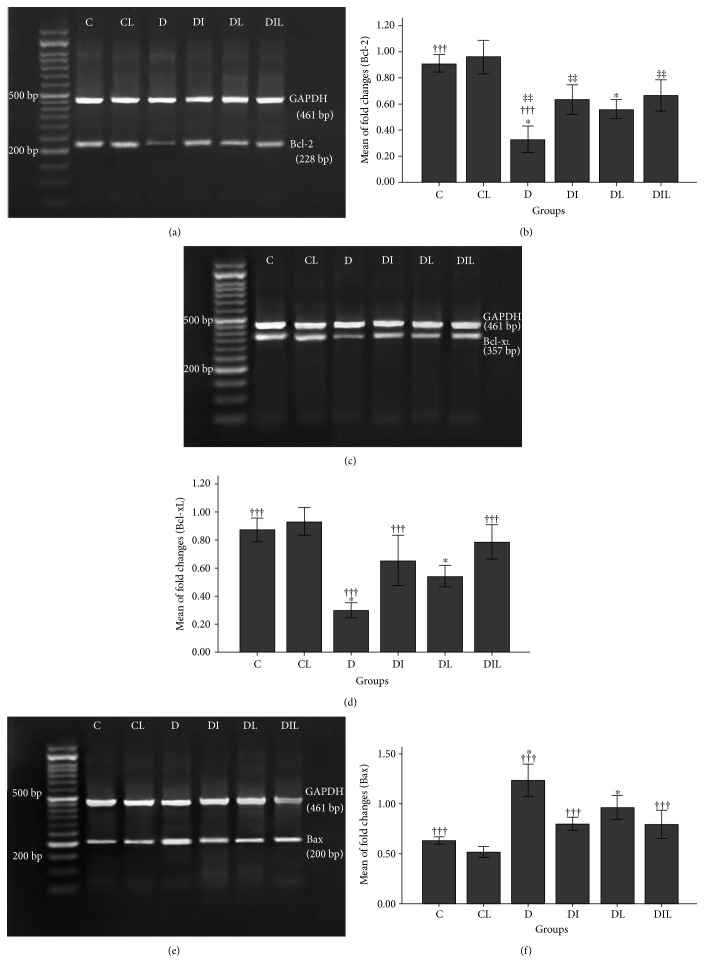
Semiquantitative RT-PCR analysis of expressions of (a) Bcl-2 gene in the hippocampus of the studied rats. Amplification of the Bcl-2 gene (228 bp) compared with that of the GAPDH gene (461 bp). (b) The effects of insulin and lycopene on expression level of Bcl-2 gene at mRNA level in the hippocampus of the studied rats. †††*p* < 0.001, comparing (expression level of Bcl-2 gene) the untreated diabetic group (D) with the control group (C). ^∗^*p* < 0.05, comparing (expression level of Bcl-2 gene) the untreated diabetic group (D) with the diabetic groups treated with lycopene (DL). ‡‡*p* < 0.01, comparing (expression level of Bcl-2 gene) the untreated diabetic group (D) with the diabetic group treated insulin (DI) and insulin and lycopene simultaneously (DIL). (c) Bcl-X_L_ gene in the hippocampus of the studied rats. Amplification of the Bcl-X_L_ gene (357 bp) compared with that of the GAPDH gene (461 bp). (d) The effects of insulin and lycopene on expression level of Bcl-X_L_ gene at mRNA level in the hippocampus of the studied rats. †††*p* < 0.001, comparing (expression level of Bcl-X_L_ gene) D with C, DI, and DIL. ^∗^*p* < 0.05, comparing (expression level of Bcl-2 gene) D with DL. (e) Bax gene in the hippocampus of the studied rats. Amplification of the Bax gene (200 bp) compared with that of the GAPDH gene (461 bp). (f) The effects of insulin and lycopene on expression level of Bax gene at mRNA level in the hippocampus of the studied rats. †††*p* < 0.001, comparing (expression level of Bax gene) D with C, DI, and DIL. ^∗^*p* < 0.05, comparing (expression level of Bax gene) D with DL. Bar graphs indicate the mean ± SEM (*N* = 8).

**Figure 3:**
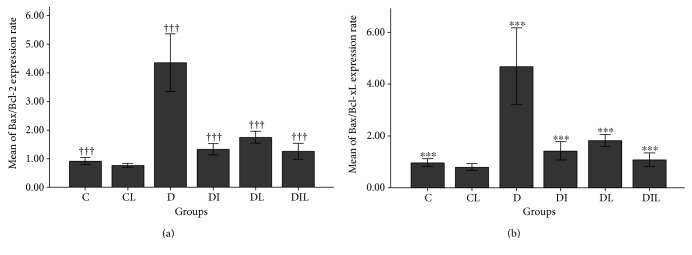
The effects of insulin or honey or both on (a) Bax/Bcl-2 ratio at mRNA level in the hippocampus of the studied rats. †††*p* < 0.001, comparing (expression level of Bax gene) the untreated diabetic group (D) with the control group (C) and the diabetic group treated with insulin (DI), lycopene (DL), and both together (DIL). (b) Bax/Bcl-X_L_ ratio at mRNA level in the hippocampus of the studied rats. ^∗∗∗^*p* < 0.001, comparing (expression level of Bax gene) the untreated diabetic group (D) with the control group (C) and the diabetic group treated insulin (DI), lycopene (DL), and both together (DIL). Bar graphs indicate the mean ± SEM (*N* = 8).

Furthermore, the *Bcl-2* gene expression level in the hippocampus of diabetic rats without treatment (D) demonstrated a significant decrease compared to that of the control group (C) (*p* < 0.001) and the diabetic group treated with insulin (DI) (*p* < 0.01) and treated with lycopene (DL) (*p* < 0.05), both alone and simultaneously (DIL) (*p* < 0.01) (Figures 2(a) and 2(b)). *Bcl-x_L_* gene expression in the diabetic group without treatment (D), however, decreased significantly compared with that in group C (*p* < 0.001), group DI (*p* < 0.001), and group DL (*p* < 0.05), both alone and simultaneously (DIL) (*p* < 0.001) (Figures 2(c) and 2(d)). *Bax* gene expression levels in the hippocampus of rats for groups C (*p* < 0.001), DI (*p* < 0.001), and DL (*p* < 0.05) both alone and simultaneously (DIL) (*p* < 0.001) showed a significant reduction in comparison to those for the nontreated diabetic (D) group (Figures 2(e) and 2(f)). The results of studying the expression of *Bax/Bcl-2* in the hippocampal region showed that this ratio had a significant increase for group D compared with that for groups C, DI, DL, and DIL (*p* < 0.001) (Figure 3(a)). As well as this, the expression of *Bax/Bcl-x_L_* in the rat's hippocampus was significantly increased in group D compared with that in groups C, DI, DL, and DL (*p* < 0.001) (Figure 3(b)).

## 4. Discussion

The results of the present study revealed that treatment of diabetic rats using alone and both of insulin and lycopene, as an antioxidant, for eight weeks led to an inhibition of histological changes in different areas of the hippocampus and decreased the expression of proapoptotic Bax gene. It also upregulated the antiapoptotic *Bcl-2* and *Bcl-x_L_* genes.

The mechanism that leads to nerve cell death is caused by hyperglycemia and has not yet been proven. However, it is hypothesized that ROS is responsible for cell death [25, 26]. Increased blood sugar stimulates an increased production of ROS. Thus, high blood sugar and increased oxidative stress are directly associated with the neurological complications of diabetes [27]. Other studies have also shown that type 1 diabetes increases oxidative stress in the hippocampus of rats and, therefore, leads to the production of ROS and causes damage to the membrane of nerve cells and mitochondrial membrane permeability, resulting in neuronal cell death [26, 27].

It has been found that the use of insulin as a blood glucose stabilizer (possibly as a neurotrophic factor) can protect neurons against apoptosis [28, 29]. Guo et al. reported that insulin can prevent the apoptosis of ganglionic neurons in diabetic rats induced by STZ [11]. Findings from previous studies have also shown that insulin, proinsulin, and insulin-like factors had similar antiapoptotic effects [28, 30]. Kooijman stated that insulin can regulate the gene expression of the *Bcl-2* family through its role as an inhibitor of apoptosis within the cells [31]. Therefore, insulin also plays a role in the stability of blood sugar levels and in inhibiting apoptosis [32].

Because numerous studies have shown that oxidative stress caused by high blood sugar can lead to the development of diabetes and its related complications, it is clear that inhibition of oxidative stress via antioxidants can be an effective method for reducing the incidence of diabetes [33–35]. In addition, experimental and clinical studies have shown that antioxidants are capable of inhibiting oxidative stress in the development of diabetes complications [36–38]. As noted previously, this research was determined to use lycopene as an antioxidant for eight weeks alone or in combination with insulin to reduce blood sugar, inhibit oxidative stress, and prevent histological changes. Overall, an increased expression of antiapoptotic *Bcl-2* and *Bcl-x_L_* genes and a decrease in the expression of the proapoptotic Bax gene were found. These findings are similar to the results of Akilandeswari and Duzguner who showed that the treatment of diabetic rats with lycopene for three weeks can significantly reduce blood sugar [39, 40]. In the study of Jade Teta et al., however, it was also found that lycopene, at a dose of 2 and 4 mg/kg for7 days, is not an antidiabetic action [41]. In addition, Zeng and colleagues showed that treatment with lycopene for three weeks, regarding the neurons of the hippocampus in rats with hyperlipidemia, plays a neuroprotective role [42].

Furthermore, the findings of this study are consistent with the findings of Qu and colleagues who found that the treatment of lycopene could reduce nerve cell death in Alzheimer's patients [43]. Yilmaz et al. also advocated that a pretreatment using lycopene increased cell survival and decreased apoptosis in the cells of the heart and kidney [44]. Compared to this study, lycopene adjusted the expression of Bcl-2, Bcl-x_L_, and Bax genes and inhibited the activity of caspase-3; findings which were consistent with Feng et al. and Sandhir et al. [45, 46]. The results of Aydin and Celik similarly showed that lycopene with an antioxidant-based effect could play an important role in inhibiting the appearance of diabetic complications [47]. The study of neuroprotective effects of lycopene in spinal cord injury in rats via antioxidative and antiapoptotic pathway which has been done by Hu and colleagues indicates that lycopene administration could improve the total antioxidant status and might have neuroprotective effects on SCI [48]. On the one hand, a research by Kuhad and colleagues on lycopene attenuates diabetes-associated cognitive decline in rats get to the result that the involvement of oxidative-nitrosative stress and peripheral inflammation in the development of cognitive impairment in diabetic animals and point towards the therapeutic potential of lycopene in diabetes-induced learning and memory impairment [21].

Lycopene, an antioxidant normally found in tomatoes [49], is the dominant carotenoid in human plasma with a half-life of 2 to 3 days [50]. Because of its strong antioxidant properties, it can be used to treat a variety of diseases, including cancer, cardiovascular disease, osteoporosis, and diabetes. Thus, lycopene can reduce oxidative stress by scavenging free radicals and preventing the damage of the essential components in cells such as lipids, proteins, and DNA [51, 52]. Lycopene obtained from food can also pass into the blood-brain barrier and enter the central nervous system. Hence, it is effective in reducing the damage caused by ROS in the brain. Overall, it is suggested that lycopene, as a neuroprotective agent, can be useful in the prevention of neurological damage during oxidative stress [41, 53]. Also, Qu and their colleagues' analysis on lycopene protects against trimethyltin-induced neurotoxicity in primary cultured rat hippocampal neurons by inhibiting the mitochondrial apoptosis pathway revealed that lycopene protects against TMT-induced neurotoxicity by inhibiting the mitochondrial apoptotic pathway. The antiapoptotic influence of lycopene on hippocampal neurons climaxes the therapeutic potential of plant-derived antioxidants contrary to neurodegenerative diseases [54].

In general, we can argue that hyperglycemia of diabetes induced by streptozotocin leads to histopathological changes in the form of apoptosis induction in the rat hippocampus. As well as this, it results in an increased expression of proapoptotic genes (*Bax*) and the downregulation of antiapoptotic genes (*Bcl-2* and *Bcl-x_L_*). In turn, treatment with insulin or lycopene, either alone or simultaneously, can inhibit apoptosis in the hippocampus by reducing the gene expression of Bax and by increasing the gene expression of *Bcl-2* and *Bcl-x_L_*. In order to prove these effects, further experimental studies, as well as clinical trials, should be proposed.

## Figures and Tables

**Table 1:** Primers used and length of segments resulting from PCR.

Genes	Primers	Product size
Bcl-2	F: 5′-CTG GTG GAC AAC ATC GCT CTG-3′	228 bp
	R: 5′-GGT CTG ACC TCA CTT GTG-3′	
Bcl-X_L_	F: 5′-AGG CTG GCG ATG AGT TTG AA-3′	357 bp
	R: 5′-TGA AAC GCT CCT GGC CTT TC-3′	
Bax	F: 5′-TTC ATC CAG GAT CGA GCA GA-3′	200 bp
	R: 5′-GCA AAG TAG AAG GCA ACG-3′	
GAPDH	F: 5′-GGC CAA GAT CAT CCA TGA CAA CT-3′	462 bp
	R: 5′-ACC AGG ACA TGA GCT TGA CAA AGT-3′	

**Table 2:** The effects of insulin or honey or both (on) blood sugar and body weight of studied rats.

Parameters	Group					
	C	CL	D	DI	DL	DIL
	Mean ± SEM	Mean ± SEM	Mean ± SEM	Mean ± SEM	Mean ± SEM	Mean ± SEM
Blood sugar (mg/dl)	107 ± 7^∗∗^	104 ± 7	412 ± 6^∗∗^	118 ± 16^∗∗^	222 ± 26^∗^	124 ± 18^∗∗^
Body weight (gr)	287 ± 13^∗∗^	284 ± 12	214 ± 17^∗∗^	243 ± 27^∗∗^	216 ± 23	299 ± 17^∗∗^

The effects of insulin or honey or both blood sugar and body weight of studied rats. Blood sugar (mg/dl): ^∗∗^*p* < 0.01, comparing the untreated diabetic group (D) with the control group (C), the diabetic group treated insulin (DI), and lycopene and insulin simultaneously (DIL). ^∗^*p* < 0.01, comparing the untreated diabetic group (D) with the diabetic group treated with lycopene (DL). Body weight (gr): ^∗∗^*p* < 0.01, comparing the untreated diabetic group (D) with the control group (C), the diabetic group treated insulin (DI), and lycopene and insulin simultaneously (DIL). No significant difference between the untreated diabetic group (D) and the diabetic group treated with lycopene (DL). Bar graph indicates the mean ± SEM.
